# Burn blister fluids in the neovascularization stage of burn wound healing: A comparison between superficial and deep partial-thickness burn wounds

**DOI:** 10.4103/2321-3868.113332

**Published:** 2015-06-26

**Authors:** Shin-Chen Pan

**Affiliations:** Department of Surgery, Section of Plastic and Reconstructive Surgery, National Cheng Kung University Medical College and Hospital, 138 Sheng Li RD, Tainan

**Keywords:** Wound healing, angiogenin, burn blister fluid, neovascularization

## Abstract

Burn wound healing is a complex and dynamic process that involves the interaction between different cell types and mediators. Neovascularization is an imperative stage of wound healing and consists of not only angiogenesis but also adult vasculogenesis. A superficial partial-thickness burn (SPTB) heals within 2 weeks without scarring. A deep partial-thickness burn (DPTB), conversely, requires 2 weeks or longer to heal and requires an aggressive treatment to prevent hypertrophic scarring. Burn blisters on the skin are a hallmark of not only SPTB but also DPTB; however, the effect of burn blister fluids on the neovascularization in these types of burns has not been fully explored. To verify this effect, the role of different burn fluids and the angiogenic factors that modulate this process are currently under investigation.

## Introduction

Burn wound healing is a dynamic and intricate process that involves various mediators and multiple cell types around the injured zone. The normal response to a break in cutaneous tissue occurs in overlapping, but biologically distinct phases. Burn wound healing can be divided into three phases, starting with inflammation and ending with matrix remodeling. The proliferative phase of wound healing begins at Day 4 and lasts until 21 days following injury.[1] The reepithelialization aspect of the proliferative phase, however, may begin within several hours following an injury and end after 2 to 3 days in the superficial burn injury wound.

**Table 1:** 

Access this article online	
**Quick Response Code**:	**Website**: www.burnstrauma.com
	**DOI**: 10.4103/2321-3868.113332

Second-degree burns are partial-thickness by definition, but they can be further categorized as either a superficial or a deep burn. Superficial partial-thickness burns (SPTB) and deep partial-thickness burns (DPTB) differ in their appearance, ability to heal, and the potential need for debridement and skin grafting. In SPTB injury, the whole layer of the epidermis is destroyed, as are various portions of the dermis. These lesions are typically pink, moist, and very painful due to the sparing damage of the nerve endings in the mid-dermis. In addition, skin blisters are often present at the injured area. Healing occurs rapidly following SPTB injury, whereby the wound quickly becomes completely reepithelialized by the migration of epithelial cells from the deeper portion of the hair follicles, as well as from the sweat and sebaceous glands to the wound site.[2] These burns generally heal within 2 weeks, and relatively little scarring occurs; however, the injured skin may result in a color change due to hyperpigmentation. This is especially known to occur in people of Asian descent. This type of injury usually has a zone of stasis and may convert to a much deeper wound, however, if the treatment is inappropriate. Unless complications occur, the optimal treatment for these wounds is to support them with regular dressing changes. In contrast to SPTB wounds, DPTB wounds involve an injury to the full thickness of the epidermis and the reticular portion of the dermis. These burns are typically dry and mottled pink or white in appearance. In addition, the heat from the burn kills the nerve endings, thus making the wound relatively insensate. Skin blisters are still present, however, making it difficult to determine the burn depth of the wound. It is therefore often difficult to distinguish between SPTB and DPTB wounds. Due to the destruction of the epithelial cells in the skin appendages, reepithelialization in these wounds is greatly retarded. Even if no infection occurs following injury, DPTB wounds still need longer than 3 weeks to heal. Due to prolongation in the inflammatory and proliferative phases, wound closure is delayed, which leads to skin scarring and contracture. Therefore, if the wound is not healed in 3 weeks, wound debridement and grafting is necessary.

## Burn blisters

Blistering may occur as a secondary event associated with a burn injury and they are found in both SPTB and DPTB wounds. Burn blisters, which are formed as a result of inflammatory changes in the early burn injury, are a physiological response that increases capillary permeability to allow for edema formation between the epidermis and the dermis.[3] The *in vivo* and *in vitro* studies that have been published regarding blister management vary in their results. These studies have shown that blisters have a stimulatory effect on wound healing due to the presence of various growth factors, while also facilitating fibroblast growth. In addition, these studies have demonstrated the detrimental aspects of blister debridement, whereby removing blisters causes a loss of the antioxidative burn blister fluid while also decreasing the circulation in an already compromised wound. Although neovascularization plays a crucial role in burn wound healing, the effect of burn blister fluids on neovascularization has not been fully explored. Burn blisters contain many growth factors, and therefore may be responsible for the neovascularization of burn wound healing.[4]

## Neovascularization in wound healing

Neovascularization includes angiogenesis and vasculo-genesis. Endothelial cells are a critical component of neovascularization and form new blood vessels through both the angiogenesis and vasculogenesis processes. Angiogenesis is the process of forming new blood vessels from pre-existing ones. The differentiation of mesodermal cells into angioblasts, cells that subsequently differentiate into endothelial cells, is believed to exclusively occur during embryonic development. This process is also called vasculogenesis. New blood vessel growth (neovascularization), however, is a process currently being re-evaluated in light of recent advances in progenitor cell biology. This concept was overturned by Asahara and colleagues, who published that purified CD34^+^ hematopoietic progenitor cells can differentiate into endothelial cells *ex vivo*.[5] These cells have been termed “endothelial progenitor cells” (EPCs) and express various endothelial cell markers. They have also been shown to incorporate into neovessels at sites of ischemia. In 1998, Shi and colleagues discovered the existence of “circulating bone marrow-derived endothelial progenitor cells” (CEPCs) in human blood.[6] Progenitor cells have been identified in the adult bone marrow and possess the ability to replace resident cells throughout the human body.[7] Although it is currently not possible to use stem cell transplantation to treat poorly healed human wounds, there are essentially three potential therapeutic strategies for the use of endothelial precursors in wound healing and tissue repair.[8] First, they may be used as a diagnostic tool to predict the risk of a tissue injury.[9] Second, endothelial precursors may be mobilized from the bone marrow into circulation using commercialized and secure growth factors, and these cells may then provide a source of vascular progenitors to help facilitate neovascularization. This has obvious therapeutic potential in instances where skin wound healing is delayed. These cells may be expanded ex vivo and subsequently transplanted to enhance neovascularization in humans, though their cell density occupies less than 0.5% of all circulating cells. The third strategy that may enhance the ability of endothelial precursor cells to aid in vascular repair is to manipulate these precursor cells *in vivo*.

## The role of burn blister fluids in burn wound neovascularization

Earlier studies that evaluated burn blister fluid emphasized keratinocyte and fibroblast proliferation.[10,11] Circulating blood cells that were observed in burn blisters, however, led us to study the role of blister fluid in the recruitment and activation of blood cells.[12] We harvested human burn fluids to study differential neovascularization from different types of burn wounds. The neovasculogenic effects of two different burn fluids were compared, and we found that burn blister fluids, especially DPTB blister fluid, presented conditions favoring the growth of endothelial cells as well as the mobilization and differentiation of circulating blood cells. Consistent with the clinical observations from our patients, it was further determined that DPTB blister fluid had an increased ability to stimulate neovascularization.[13] Superficial burn wounds generally heal with a rapid reepithelialization. Keratinocytes begin to proliferate from wound edge or hair follicle several hours after injury without much cellular migration from the new stroma.[1] Unlike superficial wounds, the formation of numerous new capillaries, either from angiogenesis or vasculogenesis, is necessary to sustain the deep wound space. Like angiogenesis, vasculogenesis is a multistep process that includes the chemoattraction, adhesion, and migration of endothelial progenitor cells to the site of injury, as well as the differentiation of endothelial progenitor cells into endothelial cells.[14] Our observations demonstrate that burn blister fluids provide a favorable environment for neovascularization in the early stage of the wound healing process.[13]

## Factors modulating the angiogenic activity of burn blister fluids

The study of cytokines involved in the wound environment is of great interest in wound-healing research. In one study that analyzed the content of wound fluids, several proteins in burn blister fluids were implicated to be involved in the wound healing process. CXCL12 was reported to have a function in the maintenance of skin integrity during an early burn injury[15] In addition, epidermal growth factor (EGF) from burn blister fluid was identified to have a role in keratinocyte proliferation.[10,16] IL-8 from burn blister fluid was also demonstrated to have the capacity to stimulate reepithelialization.[17] Furthermore, fibroblast growth factor 2 (FGF2), a potent angiogenic factor, was found to evoke a positive corneal angiogenic response not only in surgical wound fluids but also in skin graft and burn wound fluids.[18] Based on these previous findings, we are interested in studying the angiogenic contents of the different types of burn blister fluids. We will study the burn fluid contents using both human angiogenesis cytokine arrays and the Enzyme-linked immunosorbent assay (ELISA) method. Several angiogenic factors, including angiogenin, EGF, epithelial cell-derived neutrophil-activating protein-78 (ENA-78), and IL-8, have been detected in both types of burn fluids. Among them, angiogenin is most represented in blister fluids, at a concentration range of 36-574 ng per ml. Moreover, angiogenin is the only protein that displayed a significant increase in DPTB blister fluid compared with SPTB blister fluid.[19]

## The role of angiogenin in burn wound healing

Angiogenin, first discovered from the cell culture medium of colon cancer cells,[20] is a potent inducer of the angiogenic process. The surrounding tissues are known to release angiogenin when blood vessels are damaged. Angiogenin drives endothelial cells toward a stimulus to initiate the complex process of angiogenesis.[21] Angiogenesis is often induced in inflammatory joint diseases, whereby the level of angiogenin in the synovial fluid of patients with acute synovitis (104 ng/ml) has been shown to be significantly higher than in those patients with osteoarthritis (20 ng/ml).[22] The abundant expression of angiogenin in burn blister fluids indicates an association between an increased release of angiogenin with the need for neovascularization in acute burn injuries.[19]

Burn wounds remain in a dynamic state for up to 3 days after injury[23] Because SPTB wounds usually heal within days, the return of angiogenin to low levels in SPTB wounds caused by the acceleration of tissue regeneration is consistent with an earlier study in which angiogenin levels returned to baseline 3 days after injury when no further stimulation occurred.[24] In contrast, a continuously high level of angiogenin expression in DPTB fluids, even at 4 days post injury, is consistent with the high demand for neovascularization in DPTB compared with SPTB wounds.[19] Therefore, differential angiogenin expression between SPTB and DPTB wound fluids implies the different healing processes of these wounds.[19]

High serum or tissue angiogenin levels are closely related to neovascularization in various tumors.[25,26] Angiogenin was also found to contribute to the angiogenic component of tissue healing.[27] Neovascularization is a vital and fundamental requirement for burn wound healing. The observation of the increased expression of angiogenin, in relation to the development of the new vasculature in burn wounds, further supports a role for angiogenin as a burn wound neovascularization biomarker to help distinguish between SPTB and DPTB wounds.[19]

## Burn wound assessment

The early assessment of burn depth is important for burn wound management. There are many methods for assessing wound depth, including biopsy, thermography, vital dye injection, laser Doppler techniques, and bedside clinical judgment.[28] Clinical observations remain the standard for estimating clinical outcomes;[29] however, the clinical assessment of second-degree burn wounds with intact blisters has always been difficult, even for experienced surgeons. Measuring tissue perfusion in injured wounds appears to be the best approach to assess the extent of tissue damage, although laser Doppler perfusion imaging can evaluate the blood flow of burn wounds.[30] The perfusion data, however, do not always reflect burn depth and often create confusion when assessing wounds with intact blisters. The importance of using laser Doppler imaging and vimentin immunostaining was also demonstrated recently and is now considered one of the most accurate methods for the determination of burn depth.[31] In the early stage of burn wound healing, a low perfusion status of deep burn wounds induces neovascularization via hypoxia to meet the demands for regeneration. Because the angiogenic responses of SPTB and DPTB blister fluids are significantly different,[13,19] the measurement of the angiogenic contents, such as angiogenin, in burn blister fluids is a potential tool for surveying the burn wound status.

## Angiogenin in burn scar formation

DPTB wounds usually result in hypertrophic scarring. A delay in wound healing is one possible mechanism for scar formation in DPTB wounds. Understanding the role of the cytokines involved in scar formation is particularly important for scar treatment. Altered cytokine, chemokine or growth factor secretion profiles have been detected during pathologic tissue remodeling and keloid formation.[32] Angiogenin is one of these altered factors, suggesting the involvement of angiogenin as a pro-angiogenic factor in keloid formation. Hence, angiogenin neutralization at specific time points may reduce the angiogenesis of scar tissue and may therefore be useful as a potential target for scar management.

## Conclusion

The effect of burn blister fluid remains controversial. Arguments for the preservation of intact blisters center on the idea of cell proliferation, whereas the debridement of blisters has been advocated because of the perceived decreases in the oxidative damage of burned skin and wound infections that are potentially induced from chemical mediators within the burn blister. The early injury environment is robustly angiogenic, providing an early stimulus for the requisite capillary growth to support tissue repair. Thus, the fluid that accumulates at wound sites is an important reservoir of angiogenic factors that promote the neovascularization phase of the wound healing response.

**Figure 1:**
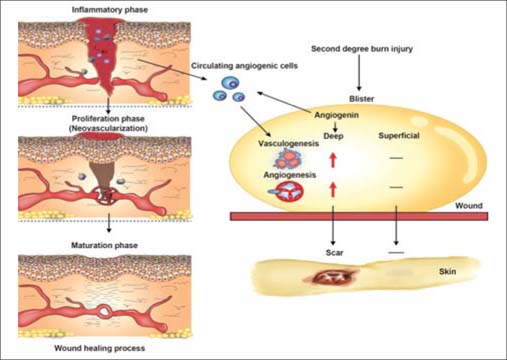
Summary of differential neovascularization between superficial and deep partial burn wounds.

In this review, we have compiled evidence for the role of burn blister fluids in neovascularization during burn wound healing. Burn blister fluids exhibit their neovasculogenic ability through not only angiogenesis but also vasculogenesis. The evidence for the angiogenic features of burn blister fluids, which may play a positive role in burn wound healing, leads us to suggest that burn blisters should remain as intact as possible. The finding that angiogenin is produced in the second-degree burn wound fluid adds this molecule to the growing list of cytokines and growth factors that may play an important role in the neovascularization of the wound healing process. Angiogenin influences the endothelial cell proliferation and differentiation of the circulating angiogenic cells and triggers *in vivo* neovascularization. Here, we provide a model to delineate how angiogenin is implicated in the differential burn wound neovascularization [Figure 1].
